# MicroRNA-448 suppresses metastasis of pancreatic ductal adenocarcinoma through targeting JAK1/STAT3 pathway

**DOI:** 10.3892/or.2022.8424

**Published:** 2022-10-11

**Authors:** Dan-Li Yu, Tao Zhang, Kun Wu, Yan Li, Juan Wang, Jun Chen, Xiao-Quan Li, Xing-Guo Peng, Jia-Ning Wang, Li-Guo Tan

Oncol Rep 38: 1075–1082, 2017; DOI: 10.3892/or.2017.5781

Following the publication of this paper, it was drawn to the Editors' attention by a concerned reader that certain of the western blot data shown in Figs. 4B and 7, the stratch-wound assay data shown in Figs. 2B and E and Fig. 6B, and the cellular images shown in Fig. 3 were strikingly similar to data appearing in different form in other articles written by different authors in different research institutions. Owing to the fact that the contentious data in the above article had already been published elsewhere prior to its submission to *Oncology Reports*, the Editor has decided that this paper should be retracted from the Journal. The authors were asked for an explanation to account for these concerns, but the Editorial Office did not receive a reply. The Editor apologizes to the readership for any inconvenience caused.

